# Supplementary material for: Distinct population code for movement kinematics and changes of ongoing movements in human subthalamic nucleus
Source: eLife. 2021 Sep 14;10:e64893. doi: 10.7554/eLife.64893 (PMC8500714; doi:10.7554/eLife.64893)
Supplement: Supplementary file 1. — Demographic characteristics of all patients and the number of single units and multi-units recorded from each. Our conclusions do not change if subjects 1, 2, or seven who contributed relatively more units than others are removed from the dataset. [file elife-64893-supp1.docx]

**Supplementary File 1: Subject demographics.** Demographic characteristics of all patients and the number of single units and multi-units recorded from each. Our conclusions do not change if subjects 1, 2, or 7 who contributed relatively more units than others are removed from the dataset.

| Patient | Age | pre-op UPDRS motor | Recording side | Single units | Multi-units | Total Units |
| --- | --- | --- | --- | --- | --- | --- |
| 1 | 61 | 36 | Right | 2 | 6 | 8 |
| 2 | 58 | 35 | Left | 2 | 4 | 6 |
| 3 | 56 | 13 | Right | 0 | 1 | 1 |
| 4 | 63 | 42 | Left | 0 | 2 | 2 |
| 5 | 51 | 42 | Left | 0 | 4 | 4 |
| 6 | 66 | 13 | Left | 1 | 1 | 2 |
| 7 | 46 | 17 | Bilateral | 3 | 9 | 12 |
| 8 | 55 | 27 | Right | 0 | 4 | 4 |
