# Supplementary material for: Distinct population code for movement kinematics and changes of ongoing movements in human subthalamic nucleus
Source: eLife. 2021 Sep 14;10:e64893. doi: 10.7554/eLife.64893 (PMC8500714; doi:10.7554/eLife.64893)
Supplement: Supplementary file 2. — Characteristics of all recorded units including the number of each trial type recorded, the classification by unit type, and the baseline firing rate (inter-trial interval). The median baseline firing rate of single- and multi-units was 32.9 Hz (IQR: 18.3–53.9) and 40.4 Hz (IQR: 20.7–82.6), respectively, suggesting that most spikes recorded from multi-units originate from one single unit. Twenty of these units had a sufficient number of left-sided and right-sided trials for independent PSTHs to be calculated for these conditions. Analyses of firing rates marginalized on trial direction (Figures 5 and 6, and Figure 5—figure supplement 2) use these units. [file elife-64893-supp2.docx]

**Supplementary File 2: Unit characteristics.** Characteristics of all recorded units including the number of each trial type recorded, the classification by unit type, and the baseline firing rate (inter-trial interval). The median baseline firing rate of single- and multi-units was 32.9 Hz (IQR: 18.3-53.9) and 40.4 Hz (IQR: 20.7-82.6), respectively, suggesting that most spikes recorded from multi-units originate from one single unit. Twenty of these units had a sufficient number of left-sided and right-sided trials for independent PSTHs to be calculated for these conditions. Analyses of firing rates marginalized on trial direction (Figs. 5, 6, and Fig. 5 – figure supplement 2) use these units.

| Unit | Subject | Location | Reach trials | Planned turn trials | Impromptu turn trials | Total trials | Unit type | Isolation | Inter-trial firing rate |
| --- | --- | --- | --- | --- | --- | --- | --- | --- | --- |
| 1 | 1 | 1 | 12 | 33 | 29 | 74 | Turn | Multi-unit | 98.6 |
| 2 | 1 | 1 | 12 | 33 | 29 | 74 | Turn | Single unit | 35.3 |
| 3 | 1 | 1 | 12 | 33 | 29 | 74 | Turn | Multi-unit | 20.0 |
| 4 | 1 | 2 | 12 | 33 | 29 | 74 | Turn | Multi-unit | 22.2 |
| 5 | 1 | 3 | 17 | 11 | 17 | 45 | Other | Multi-unit | 20.2 |
| 6 | 1 | 3 | 17 | 11 | 17 | 45 | Other | Single unit | 50.7 |
| 7 | 1 | 3 | 17 | 11 | 17 | 45 | Turn | Multi-unit | 30.1 |
| 8 | 1 | 4 | 17 | 11 | 17 | 45 | Turn | Multi-unit | 44.6 |
| 9 | 2 | 1 | 28 | 30 | 22 | 80 | Movement | Multi-unit | 10.5 |
| 10 | 2 | 1 | 28 | 30 | 22 | 80 | Movement | Single unit | 21.8 |
| 11 | 2 | 1 | 28 | 30 | 22 | 80 | Turn | Multi-unit | 28.9 |
| 12 | 2 | 3 | 47 | 29 | 31 | 107 | Other | Multi-unit | 38.6 |
| 13 | 2 | 4 | 47 | 29 | 31 | 107 | Movement | Multi-unit | 47.8 |
| 14 | 2 | 4 | 47 | 29 | 31 | 107 | Movement | Single unit | 30.6 |
| 15 | 3 | 1 | 12 | 17 | 13 | 42 | Turn | Multi-unit | 48.5 |
| 16 | 4 | 1 | 18 | 11 | 13 | 42 | Other | Multi-unit | 46.9 |
| 17 | 4 | 1 | 18 | 11 | 13 | 42 | Movement | Multi-unit | 16.1 |
| 18 | 5 | 1 | 35 | 31 | 47 | 113 | Turn | Multi-unit | 143.6 |
| 19 | 5 | 1 | 35 | 31 | 47 | 113 | Movement | Multi-unit | 14.8 |
| 20 | 5 | 2 | 10 | 29 | 27 | 66 | Movement | Multi-unit | 214.3 |
| 21 | 5 | 2 | 10 | 29 | 27 | 66 | Turn | Multi-unit | 25.7 |
| 22 | 6 | 1 | 9 | 4 | 4 | 17 | Movement | Multi-unit | 93.1 |
| 23 | 6 | 1 | 9 | 4 | 4 | 17 | Other | Single unit | 65.1 |
| 24 | 7 | 1 | 19 | 21 | 22 | 62 | Turn | Multi-unit | 9.9 |
| 25 | 7 | 1 | 19 | 21 | 22 | 62 | Turn | Multi-unit | 37.9 |
| 26 | 7 | 4 | 20 | 30 | 36 | 86 | Turn | Multi-unit | 52.6 |
| 27 | 7 | 4 | 20 | 30 | 36 | 86 | Movement | Single unit | 14.8 |
| 28 | 7 | 5 | 24 | 13 | 24 | 61 | Movement | Multi-unit | 11.3 |
| 29 | 7 | 6 | 24 | 13 | 24 | 61 | Movement | Multi-unit | 178.1 |
| 30 | 7 | 6 | 24 | 13 | 24 | 61 | Movement | Multi-unit | 82.4 |
| 31 | 7 | 7 | 23 | 26 | 25 | 74 | Turn | Multi-unit | 82.7 |
| 32 | 7 | 7 | 23 | 26 | 25 | 74 | Turn | Single unit | 57.0 |
| 33 | 7 | 8 | 23 | 26 | 25 | 74 | Movement | Multi-unit | 128.4 |
| 34 | 7 | 8 | 23 | 26 | 25 | 74 | Movement | Multi-unit | 40.4 |
| 35 | 7 | 8 | 12 | 19 | 13 | 44 | Turn | Single unit | 7.4 |
| 36 | 8 | 1 | 15 | 15 | 14 | 44 | Movement | Multi-unit | 22.9 |
| 37 | 8 | 1 | 15 | 15 | 14 | 44 | Turn | Multi-unit | 56.6 |
| 38 | 8 | 1 | 15 | 15 | 14 | 44 | Movement | Multi-unit | 82.9 |
| 39 | 8 | 2 | 15 | 15 | 14 | 44 | Turn | Multi-unit | 12.9 |
